# Electrorheological Fluids of GO/Graphene-Based Nanoplates

**DOI:** 10.3390/ma15010311

**Published:** 2022-01-02

**Authors:** Yudong Wang, Jinhua Yuan, Xiaopeng Zhao, Jianbo Yin

**Affiliations:** 1 Smart Materials Laboratory, Department of Applied Physics, School of Physical Science and Technology, Northwestern Polytechnical University, Xi’an 710129, China; 1139055802@mail.nwpu.edu.cn (Y.W.); yuanjinhua@mail.nwpu.edu.cn (J.Y.); xpzhao@nwpu.edu.cn (X.Z.); 2 Research and Development Institute of Northwestern Polytechnical University in Shenzhen, Shenzhen 518057, China

**Keywords:** graphene/GO, nanoplate, rheological properties, dielectric properties

## Abstract

Due to their unique anisotropic morphology and properties, graphene-based materials have received extensive attention in the field of smart materials. Recent studies show that graphene-based materials have potential application as a dispersed phase to develop high-performance electrorheological (ER) fluids, a kind of smart suspension whose viscosity and viscoelastic properties can be adjusted by external electric fields. However, pure graphene is not suitable for use as the dispersed phase of ER fluids due to the electric short circuit caused by its high electrical conductivity under electric fields. However, graphene oxide (GO) and graphene-based composites are suitable for use as the dispersed phase of ER fluids and show significantly enhanced property. In this review, we look critically at the latest developments of ER fluids based on GO and graphene-based composites, including their preparation, electrically tunable ER property, and dispersed stability. The mechanism behind enhanced ER property is discussed according to dielectric spectrum analysis. Finally, we also propose the remaining challenges and possible developments for the future outlook in this field.

## 1. Introduction

Smart materials refer to materials that can reversibly respond to changes in external environmental factors such as light, heat, pH, and electric/magnetic fields [1,2,3]. As a novel two-dimensional material, graphene has excellent optical, electrical, thermal, and mechanical properties [4,5,6], showing potential applications in the development of smart materials [7]. For example, when subjected to uneven forces, the honeycomb hexagonal structure on graphene’s surface can produce different deformations, resulting in different changes in the surface resistance, which enables the preparation of mechanical sensors with high sensitivity [8,9,10]. Good optical properties and high photothermal conversion efficiency also enable graphene for the preparation of self-healing materials and the drug delivery carrier of near-infrared light [10,11,12,13]. As oxide of graphene, GO can be used in thermal response drivers due to its high negative thermal expansion coefficient [14,15]. Because of the high aspect ratio, large specific surface area, and excellent physicochemical properties [4,5,6,16], recent studies show that graphene-based materials also have a potential application as dispersed phase to develop high-performance electrorheological (ER) fluids [17,18,19,20].

**Figure 1:**
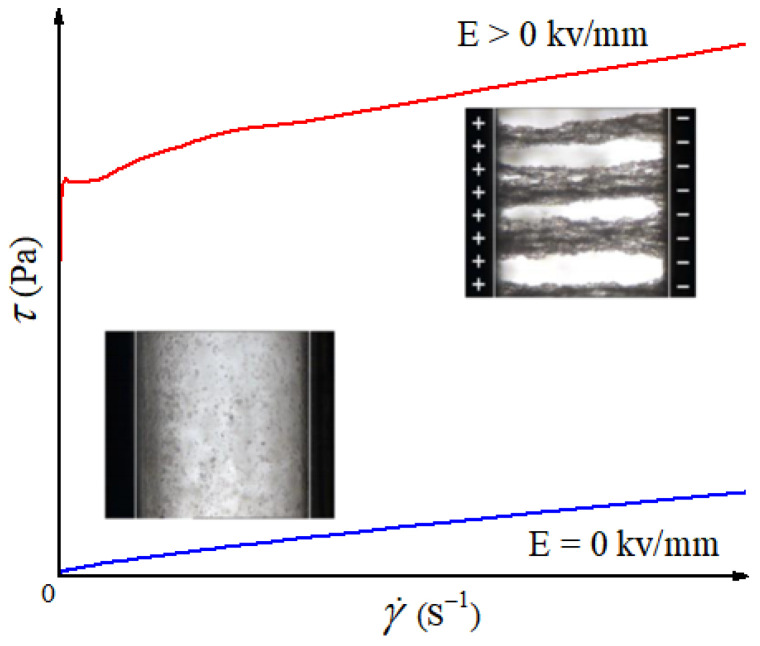
ER effect of ER fluids induced by electric field [23,24]. When E = 0 kV/mm, the particles are freely dispersed and the viscosity of fluids is low (blue line), while the particles align into a fibrous structure along the electric field direction when E > 0 kV/mm and the viscosity of fluids is significantly increased (red line).

ER fluids are a kind of suspension-like materials whose viscosity and viscoelastic properties can be adjusted by external electric fields. They are usually composed of micro/nano particles with a high dielectric constant dispersed in an insulating carrier fluid with low dielectric constant [21,22]. As shown in Figure 1 [23,24], when there is no electric field, the particles are randomly distributed in the carrier fluid and ER fluids show a Newtonian fluid state with low viscosity. When the appropriate electric field is applied, the particles are polarized and arranged in a chain/column structure across the electrode gap along the direction of the electric field, and ER fluids transform into a solid-like state possessing yield stress and high viscosity. When the electric field is withdrawn, ER fluids return to the original Newtonian fluid state. This rapid, reversible, and continuously adjustable rheology transition characteristic makes ER fluids have potentially wide applications as shock absorbing devices [25,26], smart polishing [27,28], smart lubrication [29,30], microfluid control [31,32], robotics [33,34], and medical equipment [35,36].

Since Winslow discovered the ER effect in the 1940s [37], ER fluids based on various kinds of materials have been continuously developed. The early ER materials, such as silica and starch, are water-activated [38,39,40,41]. However, the water-activated ER materials have some disadvantages, such as narrow operating temperature zone, high leaking current density, and easy corrosion of equipment. In the 1980s, anhydrous ER materials were developed to overcome the shortcomings of water-activated ER materials. The typical anhydrous ER materials include aluminosilicate, perovskite, polyaniline, polypyrrole, polyester, and the most recently reported poly(ionic liquid)s [42,43,44,45,46,47,48,49]. In particular, semi-conductive polymers and poly(ionic liquid)s have recently been frequently studied due to the advantages of soft texture, low density, little wear to the equipment, and good suspension stability. However, in addition to the inherent types of materials, the influence of the extrinsic properties of ER materials on the ER effect has also been studied. Traditional micron-level ER materials have a large particle size, which easily leads to phase separation of ER fluids, and the yield stress value induced by electric field is also relatively low. In 2003, Wen et al. developed the non-conventional ER fluids with nano-size particles as the dispersal phase and brought a new direction for ER research. The typical nano ER materials include barium titanyl oxalate (BTO), titanium oxide, and calcium titanyl oxalate (CTO) [50,51,52]. Compared with conventional ER fluids based on micro-size particles, non-conventional ER fluids based on nano-size particles have higher yield stress and suspension stability. However, the ER fluids of nano-size particles also face the problems of high zero-field viscosity and low ER efficiency, in particular at high shear rate.

Besides the size effect, the effect of particle shape on ER effect has also been considered. For example, Asano et al. [53] have proved that different morphologies of ER particles have different shear stress–shear rate characteristics. Yann et al. [54] also designed models of particles with different morphologies and studied the microstructure changes of ER fluid of elongated particles and spherical particles under electric field. In 2006, Yin et al. [55] developed a new ER fluid with titanate nanofibers. Compared with the ER fluid containing traditional granular or spherical particles, the ER fluid of one-dimensional nanofibers shows enhanced ER effect. Since then, anisotropic nanofiber ER materials have attracted wide attention and many kinds of nanofiber ER materials have been developed, such as Pb_3_O_2_Cl_2_ nanowires [56], ZnO nanowires [57], PANI nanofibers [58,59], carbon nanotubes [60], and perovskite precipitated nanorods [61,62]. The influence of aspect ratios on enhanced ER property has also been investigated. For example, Jung et al. [63] compared the ER property of PANI/mSiO_2_ composite particle suspensions with different aspect ratios, and the results showed that PANI/mSiO_2_ suspensions with higher aspect ratios had better dielectric properties and yield stress values. Yin and Xia et al. [64,65] prepared PANI and PPy fibers, respectively, and both exhibited stronger ER effects than granular PANI and PPy. Lee et al. [66] synthesized GO/SiO_2_ composite particles with different morphologies and found that fibrous GO/SiO_2_ with a higher aspect ratio has significantly enhanced ER effect compared to spherical GO/SiO_2_.

Besides one-dimensional nanofibers, two-dimensional nanoplates have also been developed as the dispersed phase of non-conventional ER fluids, because nanoplates have not only a strong long-axis polarization but also a large cross-sectional area perpendicular to the long-axis direction. In addition, compared with conventional ER materials with spherical particles, ER fluids with two-dimensional nanoplates as dispersed phase usually have a better ER effect at relatively lower particle volume fraction [67,68]. Graphene, as a two-dimensional sheet carbon material with excellent properties, has not only strong long-axis polarization and large cross-sectional area but also excellent electrical properties, which can significantly improve the polarization strength and polarization rate of its composites. As a result, recent studies have shown that graphene-based materials have potential application as dispersed phase to develop high-performance ER fluids. For example, Yin et al. [69] used conductive graphene as the core to synthesize SiO_2_/graphene composite nanoplates with strong electrical response; the rheological and dielectric performance tests indicated that SiO_2_/graphene ER materials not only had high field-induced shear stress value, but also had effective response to high frequency AC electric field.

In this review, we look critically at the latest developments of ER materials based on GO and graphene-based composites, including their preparation, electrically tunable ER property, and dispersed stability. The mechanism behind enhanced ER property is discussed according to dielectric spectrum analysis. Finally, we also propose the remaining challenges and possible developments in this field for the future outlook.

## 2. GO-Based ER Fluids

### 2.1. Pure GO

**Figure 2:**
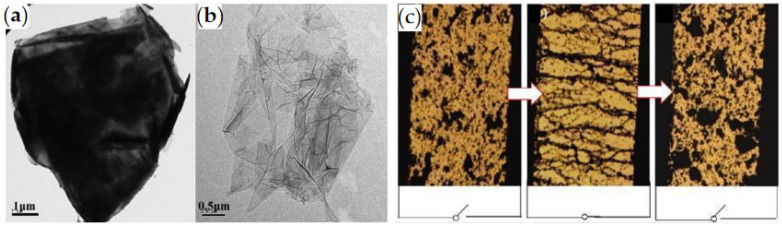
TEM images of graphene (**a**) and GO (**b**) and optical microscope photo of ER effect of GO ER fluids (**c**) [70]. It is seen that the GO particles form fibrous structure when the electric field is applied, while the particles go back to a freely dispersed state when the electric field is removed.

Pure graphene has a high leakage current density which is not suitable for direct use as dispersed particles of ER fluids. However, the surface of GO contains many oxygen-containing groups, making it insulating and polarizable, and it can be directly used as ER materials. Zhang et al. [70] prepared GO particles by using the improved Hummers method [71] and dispersed them in silicone oil, and found that the GO suspension had typical ER effect, as shown in Figure 2. However, the GO ER fluid which is directly dispersed has poor suspension stability. Hong et al. [72] prepared GO ER fluid with the high dispersion by solvent exchange method, which not only significantly improved the sedimentation stability of GO suspension but also significantly enhanced the ER effect. In addition, Dhar et al. [73] used sodium borohydride and a small amount of hydrazine hydrate to reduce GO and synthesize graphene nanogel based on polyethylene glycol, and found that graphene nanogel also had ER phenomenon.

### 2.2. Molecular-Modified GO

**Figure 3:**
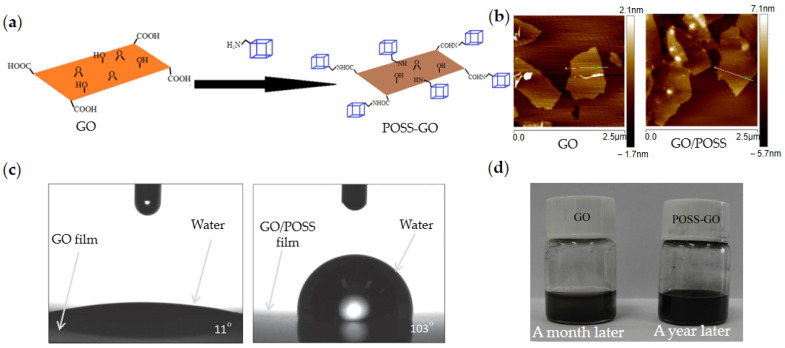
Schematic preparation of POSS/GO nanoplates by surface grafting reaction between amine functionalized POSS and oxygen-containing groups on the surface of GO (**a**) and AFM images of GO and POSS/GO (**b**) [74]; Water contact angle of GO (11°) and POSS/GO (103°) (**c**) and sedimentation performance of GO fluid and POSS/GO fluid (**d**) [74].

Although GO can be directly used as ER fluid, its surface is charged and electrophoresis easily appears under the action of the DC electric field and, as a result, the ER effect is still weak. Some researchers used molecular materials to modify the surface of GO. The modification of molecular materials can improve the ER effect and anti-settling stability of GO. As shown in Figure 3a, Li et al. [74] prepared POSS/GO nanoplates modified by POSS through surface grafting reaction between amine functionalized POSS and oxygen-containing groups on the surface of GO. Compared with pure GO, POSS/GO overcame the electrophoretic effect of pure GO and had significantly improved ER effect. The yield stress, shear storage modulus, and ER efficiency of POSS/GO ER fluid were increased by 10 times, and the hydrophilicity of GO was effectively reduced due to the notable compatibility between POSS and silicone oil (Figure 3c). The POSS/GO fluid was left for one year without sedimentation (Figure 3d). Markéta et al. [75] prepared betaine modified GO/Betaine nanoplates by silanization and thiolene click reaction, which also improved the ER effect and suspension stability of GO to a certain extent.

### 2.3. Nanoparticle-Modified GO

In order to improve the dispensability and ER effect of GO, inorganic nanoparticles were also used to modify GO. Zhang et al. [76] adsorbed modified TiO_2_ microspheres onto the surface of GO sheets by electrostatic adsorption and synthesized GO/TiO_2_ nanoplates, which showed typical ER effect. Zhang et al. [77] also synthesized Fe_3_O_4_ nanoparticles with positive charge on the surface by adjusting the pH of solution, and then prepared GO/Fe_3_O_4_ composite nanoplates with electric and magnetic double response by electrostatic adsorption method. In the composite, GO can not only be used as the supporting material but can also provide good electrical response, while Fe_3_O_4_ makes the composite exhibit good magnetic response.

### 2.4. Organic-Modified GO

**Figure 4:**
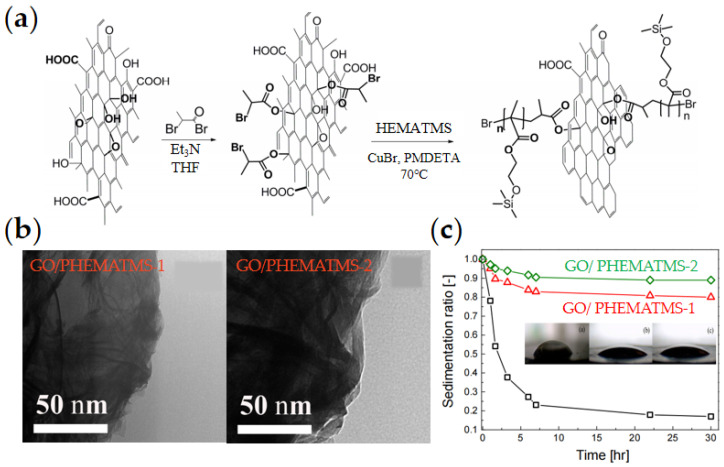
Schematic preparation of GO/PHEMATMS by SI-ATRP method (**a**) and corresponding TEM images of GO/PHEMATMS (**b**); (**c**) Sedimentation ratio of GO/PHEMATMS with different molecular weights, the molar masses of PHEMATMS-1 and PHEMATMS-2 are 12,600 g/mol and 20,400 g/mol, respectively [80].

Among various organic modification materials, poly (methacrylate) polymers are widely used. For example, Mrlík et al. [78,79] prepared GO/PGMA and GO/PBMA composites by the SI-ATRP method, respectively. Compared with pure GO, GO modified by PGMA and PBMA showed significantly enhanced ER effect. Kutalkova et al. [80] prepared two kinds of GO/PHEMATMS composites with different molar masses by the SI-ATRP method, as shown in Figure 4, and proved that the relaxation time and dielectric polarization strength of the composites had a direct influence on the ER effect. Zygo et al. [81] also prepared four kinds of composite ER materials, GO/PMMA, GO/PBMA, GO/PGMA, and GO/PHEMATMS, and all of them showed improved ER effect. It was also found that GO/PHEMATMS, which is similar to silicon-based environment, showed the best suspension stability and ER effect, where the yield stress is neat GO < PMMA < PBMA < PGMA < PHEMATMS.

**Figure 5:**
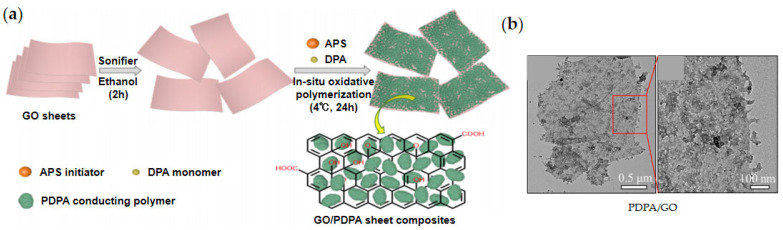
Schematic preparation of GO/PDPA by in situ oxide polymerization of DPA on the surface of GO (**a**) and corresponding TEM images of GO/PDPA (**b**) with different resolutions [82].

In addition to poly (methacrylate) polymers, Gao et al. [82] used polydiphenylamine (PDPA) to modify GO. As shown in Figure 5a, diphenylamine (DPA) monomer was first adsorbed onto the surface of GO, then APS initiator was added to initiate in situ polymerization to prepare GO/PDPA composite (Figure 5b). It was found that the introduction of GO sheets significantly enhanced the dielectric polarization of the composite and the ER effect of GO/PDPA ER fluid.

## 3. Graphene Composite ER Fluids

Although pure GO can be formulated as the dispersed phase of ER fluids and shows enhanced ER effect after modification, its ER effect is still inferior compared with one-dimensional or other nano ER fluids. Particularly, it does not effectively utilize the excellent conductivity of graphene. To simultaneously employ the excellent anisotropic morphology and electrical conductivity of graphene, the researchers have developed composite nanoplate ER fluids based on reduced graphene oxide (rGO) and graphene.

### 3.1. Graphene/Organic Composites

**Figure 6:**
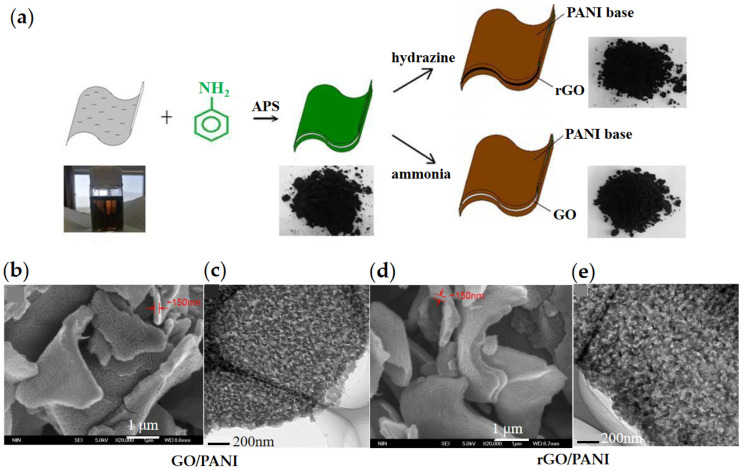
(**a**) Schematic preparation of GO/PANI by in situ polymerization of aniline on the surface of GO and the resulting rGO/PANI by hydrazine treatment of GO/PANI [85]; (**b**–**e**) SEM and TEM images of GO/PANI and rGO/PANI [83], both are ~150 nm thick.

Yin et al. [83,84] developed graphene-based composite nanoplate ER fluids by combining rGO with a semi-conducting polymer such as polyaniline and polypyrrole. As shown in Figure 6a, they first prepared PANI-coated GO composite nanoplates by the in situ polymerization method and then skillfully reduced PANI from a conductive state to an insulating state and reduced GO from an insulating state to a conductive rGO state by hydrazine treatment, thus forming composite nanoplates with a conductive rGO core and insulating PANI shell [85]. Figure 6b shows the SEM and TEM images of rGO/PANI composite. The structure effectively utilizes the morphology and electrical performance of rGO to improve the dielectric constant of the composite, from 11.2 (pure PANI) to 82.9 (rGO/PANI). Rheological results showed that rGO/PANI composite nanoplates had significantly enhanced ER effect compared with pure PANI.

Yin et al. [84] also synthesized rGO/polypyrrole (rGO/PPy) core-shell structured nanoplates by in situ polymerization and thermal reduction and found that the introduction of rGO as a support core also significantly enhanced the ER properties of composite nanoplates. Wang et al. [86] reduced GO/poly(ethylaniline) (GO/PEANI) with different concentrations of hydrazine to control the electrical properties of PEANI shell and GO core and studied the relationship between the matching degree of electrical properties of core-shell structure and ER property.

In the above methods, by controlling the reduction conditions, not only can the doping degree of conductive polymer be adjusted but also the number of oxygen-containing functional groups on the surface of GO can be controlled. As a result, the ER effect of composite nanoplates can be optimized. It provides a new strategy for developing high-performance composite ER materials with controllable electrical properties and proficient ER effect [86,87].

In addition to semi-conducting polymers, chitosan has also been used to insulate graphene in the development of composite ER materials. For example, Hu et al. [88] prepared chitosan modified graphene two-dimensional composites by microwave assisted synthesis and found typical ER effect and improved suspension stability.

### 3.2. Graphene/SiO_2_

**Figure 7:**
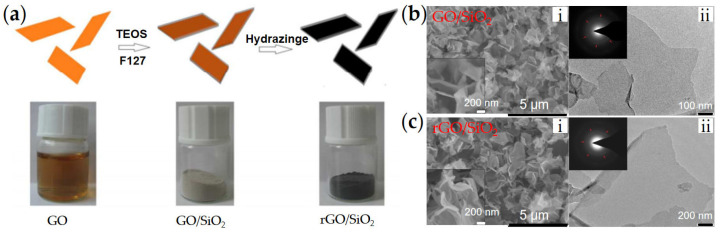
(**a**) Schematic preparation of rGO/SiO_2_ nanoplates by TEOS hydrolysis on the surface of GO and hydrazine treatment; (**b**) SEM image (i), TEM image (ii) and SAED (inset) of GO/SiO_2_; (**c**) SEM image (i), TEM image (ii) and SAED (inset) image of rGO/SiO_2_ [68].

In order to achieve an effective electrically insulating effect for graphene and improve the wettability of the graphene to carrier liquid (silicone oil), Li et al. [68] synthesized SiO_2_-coated rGO two-dimensional composite nanoplates (rGO/SiO_2_) by a simple wet chemical method, as shown in Figure 7a. SiO_2_ has good compatibility with silicone oil and can provide an insulating shell for highly conducting rGO. The high electrical conductivity of the rGO core enhances the interfacial polarization rate of rGO/SiO_2_ and makes the rGO/SiO_2_ have a respectable ER effect under even high frequency AC electric field. In addition, graphene/mesoporous SiO_2_ composite nanoplates were also synthesized by Yin et al. [69]. The coating of mesoporous SiO_2_ also provides insulation for graphene and further increases the contact area and compatibility between composite nanoplates and carrier liquid, greatly improving the dispersion stability of composite nanoplates measured by direct observation.

### 3.3. Graphene/C

Carbon-containing materials are a large category of ER materials with excellent performance. Yin et al. [89] used vacuum annealing GO/PANI composite nanoplates to transform the GO core into a conductive graphene core and the PANI shell into an insulating nitrogen-rich amorphous carbon to get graphene/C composite nanoplates. The graphene/C composite nanoplates maintain a two-dimensional sheet morphology with a high aspect ratio and excellent electrical properties of graphene. Compared with pure carbon particles, the yield stress of graphene/C ER fluid is increased by three times.

### 3.4. LDH/Graphene

**Figure 8:**
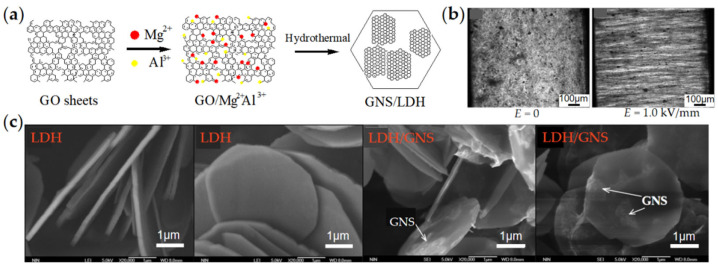
(**a**) Schematic preparation process of LDH/graphene (LDH/GNS) nanoplates by one-pot hydrothermal reaction of Mg(NO_3_)2·6H_2_O, Al(NO_3_)3·9H_2_O, and hexamethylenetetramin on GO surface; (**b**) Optical microscope photo of ER effect of LDH/GNS; (**c**) SEM images of LDH/GNS showing a smaller area of conductive GNS adsorbed on a larger area of LDH [90].

Graphene in the above graphene/GO-based two-dimensional composite nanoplates usually acts as a core or supporting material. In 2014, Dong et al. [90] developed a new type of two-dimensional graphene-based composite ER material, which is different from the previous coating structure. As the schematic preparation and SEM images in Figure 8 show, this composite nanoplate is composed of a smaller area of conductive graphene adsorbed on a larger area of layered double-hydroxide (LDH/GNS). When the electric field is applied, LDHs are arranged into chains, but graphene does not contact them directly, which greatly limits the large leaking current density. However, the high conductivity of graphene significantly enhances the dielectric polarization of composite nanoplates and improves the interaction between composite nanoplates. As a result, compared with pure LDH, LDH/GNS showed a significantly enhanced ER effect.

## 4. ER Property of Graphene and GO-Based Composites

### 4.1. Rheological Property

**Figure 9:**
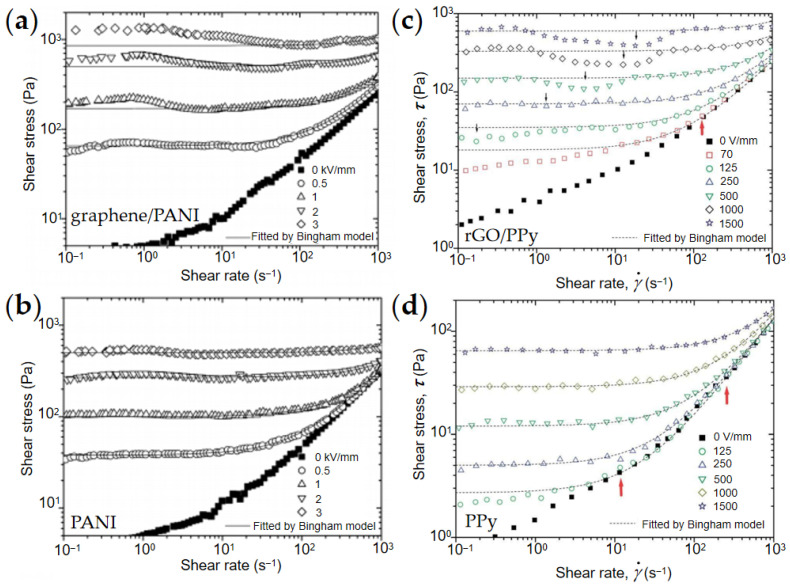
Flow curves of shear stress–shear rate of ER fluids under DC electric field. (**a**) graphene/PANI and (**b**) PANI [83]; (**c**) rGO/PPy and (**d**)pure granular PPy [84].

Rheological property is one of the most important properties of ER fluids. The relationship between shear rate and shear stress obtained in shear tests reflects the rheological behavior of ER fluids under different electric fields. As shown in Figure 9, the ER fluids behave like Newtonian fluid without an applied electric field, and the shear stress increases almost linearly with the increase in shear rate. When the electric field is applied it induces polarization of particles, and the ER fluids behave like Bingham fluid and have a certain yield stress that increases with the increase in electric field intensity. Figure 9 compares the ER properties of typical graphene composite nanoplate ER fluids. Compared with pure PANI, PPy, and rGO/PPY, composites with conductive graphene or rGO as core showed significantly enhanced electric field-induced shear stress, indicating that the introduction of graphene materials can significantly improve the ER effect [83,84,89].

**Figure 10:**
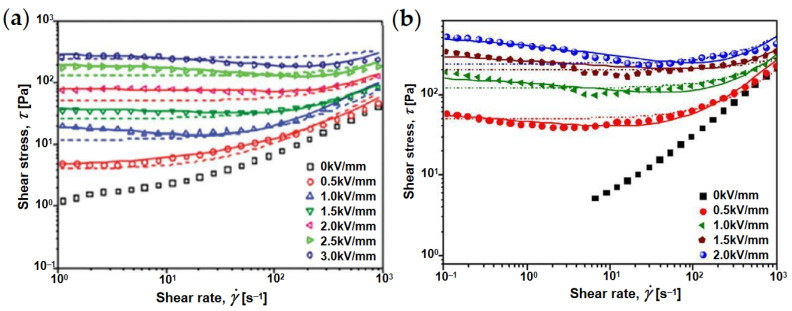
Flow curves of shear rate–shear stress of ER fluids of GO/SiO_2_ (**a**) and GO/PANI (**b**); The solid and dashed lines are fitted by CCJ and Bingham models, respectively [93,94].

However, compared with pure nanoplates, the ER behavior of ER fluids of composite nanoplates is more complex, as shown Figure 9. It can be seen that the flow curves of pure nanoplates can be fitted with a simple Bingham equation (Equation (1)) [91], while those of composite nanoplates represent a departure from the Bingham model [92]. As shown in Figure 10, Zhang et al. [93,94] compared the fitting of shear rate–shear stress relationship curve of composite nanoplates by CCJ model (Equation (2)) and the Bingham model, and found that the CCJ model could better fit the ER behavior of composite nanoplates. This is because the introduction of graphene materials not only enhances the dielectric polarization intensity but also significantly changes the dielectric polarization rate of the composites due to the high conductivity of graphene.

(1) $$\tau =\tau_{d}+\eta_{0}\dot{\gamma }$$

(2) $$\tau =\frac{\tau_{d}}{1+{\left(t_{1}\dot{\gamma }\right)}^{\alpha }}+\eta_{\infty }\left(1+\frac{1}{{\left(t_{2}\dot{\gamma }\right)}^{\beta }}\right)\dot{\gamma }$$

where τ is shear stress, $\tau_{d}$ is dynamic yield stress, $\eta_{0}$ stands for zero electric field viscosity, $\dot{\gamma }$ is shear rate, $\eta_{\infty }$ is the viscosity of ER fluid at high shear rate, α and β are related to the shear stress variation in the region of low shear rate and high shear rate, respectively;$t_{1}$ and $t_{2}$ are the time exponent of shear stress change.

**Figure 11:**
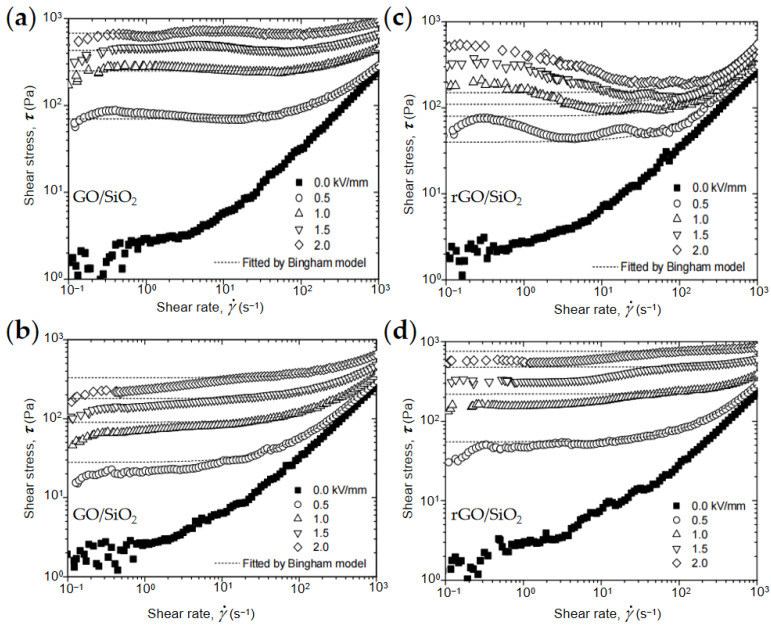
Flow curve of shear stress–shear rate of ER fluids of GO/SiO_2_ under DC electric field (**a**) and under 1000 Hz of AC electric field (**b**); Flow curve of shear stress–shear rate of ER fluids of rGO/SiO_2_ under DC electric field (**c**) and under 1000 Hz of AC electric field (**d**) [68].

In order to study the influence of graphene core on the dielectric polarization rate and resulting ER property of composite nanoplates, Yin’s group further compared the flow behaviors of GO and graphene-based composites under different DC and AC electric fields. Figure 11 shows the flow curves of different ER fluids under the DC electric field and AC electric field [68]. It found that using GO as a core can induce respectable ER effect to the DC electric field (Figure 11a), while using highly conductive rGO as a core can induce good ER effect to the high frequency AC field (Figure 11d). This is caused by the different electrical conductivity of GO and rGO, resulting in different dielectric polarization rates.

### 4.2. Yield Stress

**Figure 12:**
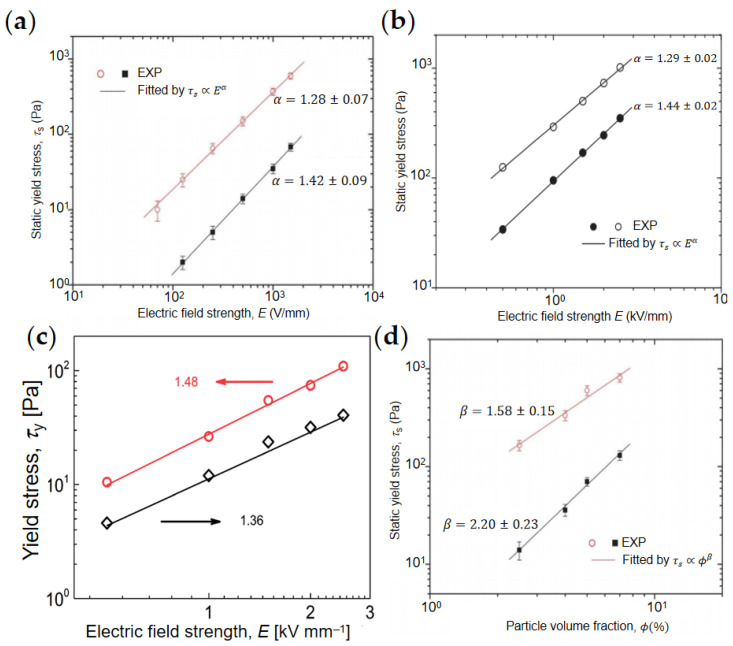
Yield stress–electric field intensity relationship of ER fluids of rGO/PPy (open circle point) and pure granular PPy (solid square point) (**a**) [84], graphene-supported carbonaceous sheets (open points) and pure carbonaceous particles (solid points) (**b**) [89], neat GO (**◇**) and GO-PBMA (**○**) (**c**) [79]; Yield stress-particle volume fraction relationship of ER fluids of rGO/PPy (open circle point) and pure granular PPy (solid square point) (**d**) [84].

The yield stress of ER fluids includes dynamic yield stress $\tau_{d}$ and static yield stress $\tau_{s}$. $\tau_{d}$ refers to the minimum stress required to maintain the continuous flow of ER fluids when the electric field and shear field work together. $\tau_{s}$ refers to the minimum stress required to make the solidified ER fluids start to flow. $\tau_{d}$ value can usually be obtained by CCJ and Bingham model fitting, which is important to characterize the strength of ER effect. Graphene/GO-based composite ER materials can usually significantly enhance the yield stress. Meanwhile, there is a power-law relationship between the yield stress $\tau_{d}$ and the electric field intensity *E*, $\tau_{d\;}\propto E^{\alpha }$. As shown in Figure 12a,b, Yin et al. [84,89] noted that the α value of nanoplate ER fluids was less than that of conventional ER fluids of spherical particles. This may be because of the morphology effect. In Figure 12c, Mrlik et al. [79] also used the power-law model to fit the yield stress of GO and GO-PBMA and found that the α value of GO-PBMA ER fluid was 1.48, which is larger than 1.36 of pure GO ER fluid.

In addition, as shown in Figure 12d, Yin et al. [84] fitted the relationship between particle concentration and yield stress by formula $\tau_{s\;}\propto \phi^{\beta }$, and found that the yield stress of rGO/PPy and pure PPy increased with the increase in particle volume fraction. However, the index of rGO/PPy (β=1.58±0.15) was smaller than that of pure PPy (β=2.20±0.23), indicating that rGO/PPy with plate structure had better ER effect compared to granular PPy at low particle concentration. This is also because of the morphology effect.

### 4.3. Dynamic Viscoelasticity

**Figure 13:**
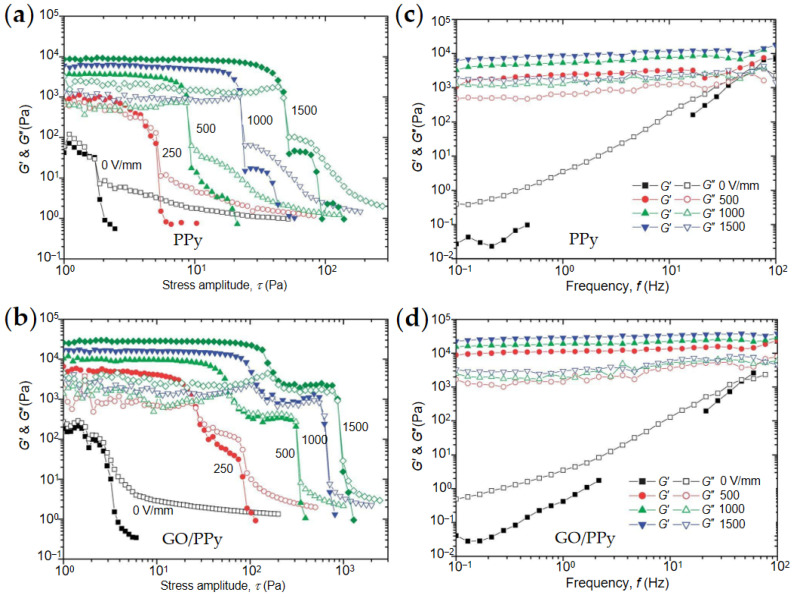
Storage modulus $G^{\prime }$ (solid points) and the loss modulus G″ (open points) as a function of oscillation stress (**a**,**b**), and storage modulus $G^{\prime }$ (solid points) and the loss modulus G″ (open points) as a function of frequency (**c**,**d**) for ER fluids: (**a**) PPy, (**b**) GO/PPy, (**c**) PPy, and (**d**) GO/PPy [84].

The dynamic viscoelasticity is also an important parameter to evaluate ER effect. The complex modulus G* of ER fluids can be defined by $G*=\tau_{0}/\gamma_{0}$, where $\tau_{0}$ is the amplitude of shear stress, $\gamma_{0}$ is the amplitude of shear strain [95], G* can be expressed as $G*=G^{\prime }+jG\prime \prime$, where the storage modulus $G^{\prime }$ and loss modulus G″ can be obtained through the oscillation mode test [96]. As shown in Figure 13, Yin and Li et al. [74,84] obtained the relationship between the storage modulus and loss modulus of ER fluids with the oscillation frequency/stress through the oscillation test. It can be seen from Figure 13 that when the electric field strength is off, $G^{\prime }$ of the ER fluids is less than G″, indicating the ER fluids are in a liquid-like state. When the electric field is on, $G^{\prime }$ of ER fluids is greater than G″, indicating that ER fluids are in a solid-like state. Compared with ER fluid of pure granular PPy, the ER fluid of rGO/PPy composite nanoplates has a higher storage modulus, which indicates that the introduction of rGO improves ER effect. In addition, from Figure 13a, it is seen that the yield of solidified ER fluid is significantly different between pure PPy and PPy/rGO nanoplates. The introduction of rGO enables GO/PPy ER fluid to have a two-step yield process.

### 4.4. Reversibility of ER Effect

**Figure 14:**
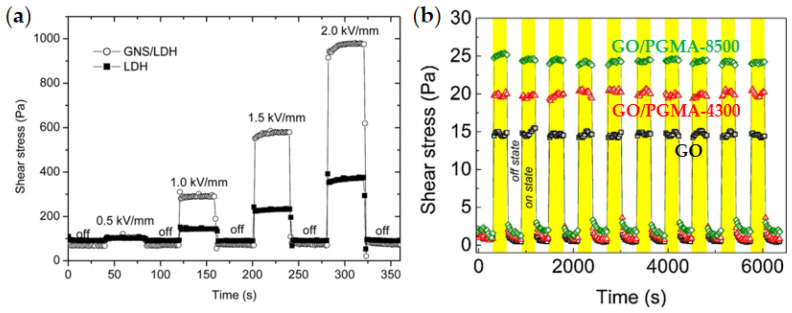
The on/off field response for ER fluids: (**a**) GNS/LDH and LDH [90]; (**b**) GO/PGMA and GO [78].

The reversibility of the ER effect of ER fluids is also important for real application. In Figure 14a, Dong et al. [90] tested the electrical responsiveness of LDH and GNS/LDH composite ER materials by alternately turning on/off the electric field and found that GNS/LDH still had reversible electrical responsiveness after turning the electric field on/off many times. With the increase in the electric field’s intensity, the shear stress of GNS/LDH composite particles also increased significantly. The shear stress of GNS/LDH composite is 2.5 times that of the pure LDH. Mrlík et al. [78] also tested the reversibility of the electric response of GO/PGMA and found that, after alternating the electric field GO/PGMA many times, the composite ER fluid could still maintain stable and reversible electric response performance, as shown in Figure 14b. At the same time, compared with pure GO, the GO/PGMA composite ER fluid exhibited enhanced shear stress.

## 5. Dielectric Properties

Dielectric polarization is the origin of ER effect. In ER fluids, the dispersed particles and the insulating carrier fluid usually have a large difference in dielectric constant and conductivity. When an electric field is applied, the positive and negative charges in the dispersed particles move to both ends, respectively, forming dipoles. Under the action of dipole–dipole electrostatic force, the particles are arranged into a chain structure along the direction of the electric field, which increases the apparent viscosity of ER fluids, or the so-called ER effect. 

Generally, in order to quantitatively describe the dielectric properties of ER fluids, the dielectric function (Equation (3)) containing Cole–Cole term and DC conductance term is usually used to fit the dielectric data [87,97,98,99]:

(3) $$\varepsilon^{*}\left(\omega \right)=\varepsilon^{\prime }+i\varepsilon^{\prime \prime }=\varepsilon_{\infty }^{\prime }+\frac{\Delta \varepsilon^{\prime }}{1+{\left(i\omega \lambda \right)}^{\beta }}+i\frac{\sigma }{\varepsilon_{0}\omega }$$

where $\Delta \varepsilon^{\prime }=\varepsilon_{0}^{\prime }-\varepsilon_{\infty }^{\prime }$ ($\varepsilon_{0}^{\prime }$ and $\varepsilon_{\infty }^{\prime }$ represent the dielectric constants corresponding to the upper and lower limits of the angular frequency in the dielectric relaxation distribution), ω is angular frequency, σ is electrical conductivity, λ is the relaxation time, and $\lambda =1/\omega_{max}$ ($\omega_{max}$ represents the angular frequency corresponding to the loss peak), β represents the dispersion index of relaxation time. 

**Figure 15:**
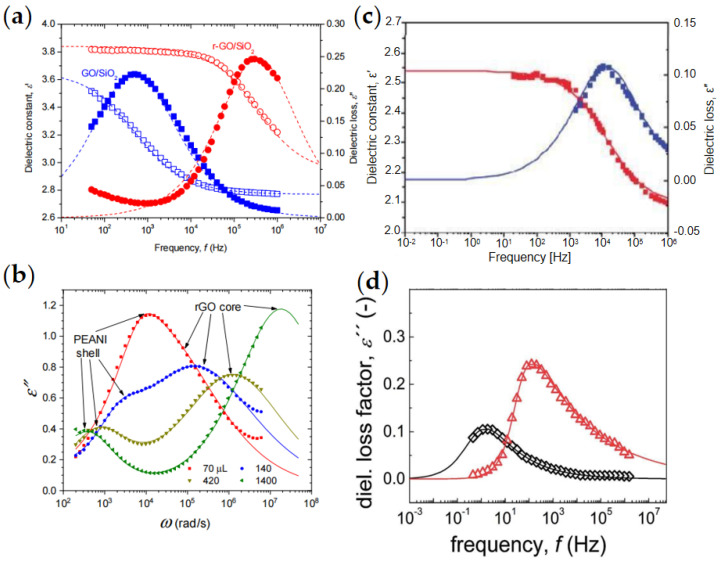
Dielectric spectra of ER fluids. (**a**) GO/SiO_2_ and rGO/SiO_2_ [68]; (**b**) PEANI/rGO with different reduction degree [86]; (**c**) GO [70]; (**d**) GO (**◇**), and GO/PBMA (**△**) [79].

The conductivity of pure graphene is too high to produce effective dielectric polarization. However, using graphene as a core and semiconducting polymer or inorganic oxide as a shell can form core/shell structured dielectric nanoplates. Meanwhile, the high conductivity of graphene can significantly enhance the dielectric polarization of the composite. In Figure 15a, Li et al. [68] used hydrazine to reduce GO/SiO_2_ composites to get rGO/SiO_2_ with high conductivity of rGO core. The rGO core endowed the rGO/SiO_2_ composite with faster dielectric polarization rate and stronger polarization strength, which made rGO/SiO_2_ nanoplates have respectable ER effect under even high-frequency AC electric field. In Figure 15b, Wang et al. [86] prepared GO/PEANI composite and adjusted the conductivity of GO core and PEANI shell by reduction treatment with different concentration of hydrazine solution. Under the DC electric field, they found that composite particles with matching electrical properties had more stable ER properties. When the relaxation time of dispersed particles is in the range of 1.6 × 10^−3^–1.6 × 10^−6^ s [24,100,101,102], the particles have an appropriate polarization rate. Under the combined action of electric field and shear field, the particles can produce a sufficient polarization rate and stable interaction force between particles.

As the oxide of graphene, GO contains a large number of oxygen-containing groups on its surface, which form defects on the surface of graphene to restrict the movement of carriers and make GO have strong dielectric polarization properties. At the same time, both electrical conductivity and dielectric polarization properties are adjustable by reduction treatment. As shown in Figure 15c, Zhang et al. [70] obtained the dielectric constant $\varepsilon^{\prime }$/dielectric losses $\varepsilon^{\prime \prime }$-frequency relationship of pure GO ER fluid and found that pure GO had a notable dielectric relaxation peak, which corresponds to the typical ER effect of pure GO ER fluid. Mrlik et al. [79] compared the dielectric properties of pure GO and GO/PBMA composite ER fluids. It was observed from Figure 15d that the GO/PBMA composite sheet material had an obviously higher relaxation peak and shorter relaxation time, thus, the GO/PBMA composite nanoplates also showed a better ER effect than pure GO.

Finally, Table 1 summarizes the properties of two-dimensional sheet graphene/GO-based composite ER materials. 

## 6. Conclusions

In this review, we summarized the recent advance of graphene/GO-based nanoplates as high-performance ER materials, including the preparation, rheological properties, dielectric properties, and dispersed stability. Pure GO can be directly used as an ER material due to its low conductivity. However, its ER effect needs to be improved by surface modification. To improve ER effect, researchers have developed molecular-, polymer-, and nanoparticle-modified GO nanoplates by different preparation methods. These modifications not only improve the ER properties but also increase the dispersed stability of GO in oil carrier liquid. Pure graphene cannot be directly used as ER material due to its high conductivity. However, by using graphene as a supporting core, researchers have developed many kinds of novel core-shell structured nanoplates for use as ER materials, such as graphene/PANI, graphene/PPy, graphene/SiO_2_, graphene/C, etc. Graphene core not only induces anisotropic two-dimensional nanoplate morphology but also enhances the dielectric polarization of composites, while an organic or inorganic shell well limits the high leakage current density of graphene. As a result, the composites show a strong ER effect, in particular for high frequency AC electric fields. Despite the significant advances that have been made in this area, there remain several challenges that require future work at this frontier.

(1). In the material preparation, due to the problems of dispersion and aggregation of GO/graphene, the repeatability of the synthesis of GO/graphene-based nanoplates is sometimes relatively low. This restrains the mass preparation and wide application of graphene-based ER materials.
(2). The material performance of GO/graphene-based nanoplate ER materials is relatively singular, and there is little development of multifunctional smart GO/graphene-based ER materials. Multifunctional GO/graphene-based ER materials may be more suitable for the requirements of future technologies for smart materials, for example, to develop GO/graphene-based nanoplate ER materials with dual responses to electric and magnetic fields.
(3). In applications, present research on GO/graphene-based ER materials is usually limited to field-controlled rheological properties. In addition to electrical properties, graphene also has excellent thermal, optical, and mechanical properties, which are conducive to expanding the application fields of GO/graphene-based ER materials. For example, by combining ER effect and the high thermal conductivity of graphene, it is possible to realize the electric field control of heat conduction.

## Figures and Tables

**Table 1:** The properties of GO/graphene-based nanoplate ER materials.

	Material Category	Material	Method	Size	Particle Concentration	Yield Stress τ (Pa)	$\varepsilon^{\prime }$	$\Delta \varepsilon_{\infty }$	λ (s)	σ (S/cm)	Sedimentation Ratio
GO-based composite	Pure GO	GO [70]	Direct dispersion	~5 µm	5 wt%	~180 (2.5 kV/mm)	2.54	0.45	1.3 × 10^−5^	-	-
		GO [72]	Solvent exchange	-	5 wt%	~270 (5 kV/mm)	-	-	-	1.0 × 10^−7^	~95%
	Molecular/GO	POSS/GO [74]	Electrostatic adsorption	Thickness ~3.5 nm	-	~600 (3 kV/mm)	-	-	-	-	One year without settlement
		Betaine/GO [75]	Silanization and thiolene click reaction	-	1 wt%	~97 (3 kV/mm)	4.98	1.73	0.04	6.22 × 10^−8^	-
	Nanoparticle/GO	TiO_2_/GO [76]	Electrostatic adsorption	-	15 wt%	-	-	-	-	5.5 × 10^−10^	-
		Fe_3_O_4_/GO [77]	Electrostatic adsorption	-	15 wt%	-	-	-	-	10^−7^	-
	Organic/ GO	PGMA/GO [78]	SI-ATRP	-	-	~100 (2 kV/mm)	5.39	1.81	0.009	6.1 × 10^−7^	90%
		PBMA/GO [79]	SI-ATRP	-	5 wt%	~110 (2.5 kV/mm)	4.35	1.37	0.005	6.0 × 10^−7^	60%
		PHEMATMS/GO [80]	SI-ATRP	-	5 wt%	200 (3 kV/mm)	-	-	0.002	6.0 × 10^−6^	90%
		PMMA/GO [81]	SI-ATRP	-	5 wt%	~68 (2.5 kV/mm)	-	-	-	6.3 × 10^−8^	85%
		PDPA/GO [82]	In situ polymerization	-	-	76 (1.8 kV/mm)	4.45	1.54	6.0 × 10^−6^	8.0 × 10^−8^	-
Graphene/rGO-based composite	Graphene/Organic	PANI/rGO [83]	In situ polymerization	Thickness 40–75 nm	10 vol%	1220 (3 kV/mm)	82.9	-	-	4.0 × 10^−9^	-
		Ppy/GO [84]	In situ polymerization	Thickness ~25–70 nm	5 vol%	~600 (1.5 kV/mm)	~5.1	-	-	9.6 × 10^−9^	-
		PEANI/GO [85]	In situ polymerization	3–10 μm, Thickness ~200 nm	4.5 vol%	~420 (1.5 kV/mm)	-	5.02	3.0 × 10^−5^	1.09 × 10^−9^	-
		Chitosan/graphene [88]	Electrostatic adsorption assisted by microwave	-	0.33 wt%	-	-	-	-	-	24h without settlement
	Graphene/SiO_2_	SiO_2_/rGO [68]	Wet chemical method	~1.5 μm, Thickness ~30 nm	3 vol%	578 (2 kV/mm)	3.83	0.92	4.5 × 10^−7^	1.7 × 10^−9^	-
		Graphene/mSiO_2_ [69]	Sol-gel	~2 µm, Thickness ~20 nm	5 vol%	850 (1.5 kV/mm)	~6.5	-	3.2 × 10^−7^	2.8 × 10^−9^	~95%
	Graphene/C	Graphene/C [89]	Vacuum annealing	Thickness ~240 nm	10 vol%	1100 (2.5 kV/mm)	~5.6	-	4.0 × 10^−5^	3.3 × 10^−8^	-
	Graphene/LDH	Graphene/ LDH [90]	One-pot hydrothermal method	3–5 µm, Thickness 50–100 nm	10 wt%	1000 (2.0 kV/mm)	5.85	2.55	1.2 × 10^−7^	8.0 × 10^−9^	-

## Data Availability

Not applicable.
